# Vasoactive intestinal peptide excites GnRH neurons via KCa3.1, a potential player in the slow afterhyperpolarization current

**DOI:** 10.3389/fncel.2024.1354095

**Published:** 2024-04-03

**Authors:** Stephanie Constantin, Clarisse Quignon, Katherine Pizano, David M. Shostak, Susan Wray

**Affiliations:** Cellular and Developmental Neurobiology Section, National Institute of Neurological Disorders and Stroke/National Institutes of Health, Bethesda, MD, United States

**Keywords:** VIP, GnRH neurons, slow afterhyperpolarization, KCa3.1, circadian rhythms, reproduction

## Abstract

Vasoactive intestinal peptide (VIP) is an important component of the suprachiasmatic nucleus (SCN) which relays circadian information to neuronal populations, including GnRH neurons. Human and animal studies have shown an impact of disrupted daily rhythms (chronic shift work, temporal food restriction, clock gene disruption) on both male and female reproduction and fertility. To date, how VIP modulates GnRH neurons remains unknown. Calcium imaging and electrophysiology on primary GnRH neurons in explants and adult mouse brain slice, respectively, were used to address this question. We found VIP excites GnRH neurons via the VIP receptor, VPAC2. The downstream signaling pathway uses both Gs protein/adenylyl cyclase/protein kinase A (PKA) and phospholipase C/phosphatidylinositol 4,5-bisphosphate (PIP_2_) depletion. Furthermore, we identified a UCL2077-sensitive target, likely contributing to the slow afterhyperpolarization current (I*_*AHP*_*), as the PKA and PIP_2_ depletion target, and the KCa3.1 channel as a specific target. Thus, VIP/VPAC2 provides an example of Gs protein-coupled receptor-triggered excitation in GnRH neurons, modulating GnRH neurons likely via the slow I*_*AHP*_*. The possible identification of KCa3.1 in the GnRH neuron slow I*_*AHP*_* may provide a new therapeutical target for fertility treatments.

## Introduction

Fertility relies on multiple signals which are ultimately integrated by gonadotropin releasing hormone (GnRH) neurons, the last central nervous system component driving the hypothalamic-pituitary-gonadal axis (HPG) and thus reproduction. Synchronizing reproductive physiology with daily and seasonal environmental cues is a necessity to ensure breeding success and offspring survival. Certainly, the duration of daylight and onset of light or dark (Hastings et al., 1985; Wright et al., 2013) influences the release of GnRH and gonadotropins. The suprachiasmatic nucleus (SCN) conveys time-of-the-day signals to the HPG (Wiegand et al., 1980; Li et al., 2023). Human studies have shown that central pubertal activation is accompanied by a nocturnal rise in luteinizing hormones (LH) concentrations, in both males and females (Demir et al., 2023) and that disruption of daily cycles (e.g., chronic shift work) impacts both male and female reproduction and fertility (Moralia et al., 2022 for review), pinpointing the importance of understanding how the SCN message is integrated by the HPG axis.

Vasoactive intestinal peptide neurons within the SCN project to GnRH neurons in both male and female animals [rodents: (Beek et al., 1994; Van der Beek et al., 1997; Horvath et al., 1998); non-human primates: (Abizaid et al., 2004)]. *In vivo* studies support a role for VIP signaling in the generation and/or timing of the GnRH/LH surge in females, with infusion of VIP antisense into the SCN or specific ablation of SCN VIP neurons attenuating the steroid-induced LH surge (Harney et al., 1996; Kahan et al., 2023), and reproductive behavior in males (Kukino et al., 2022). *In vitro*, VIP stimulates GnRH release (Samson et al., 1981) and in *ex vivo* preparations, VIP increases GnRH cell firing rate in both male and female mice (Piet et al., 2016), though the response is time-of-the-day and estradiol (E2)-dependent in females to optimize the pre-ovulatory surge (Christian and Moenter, 2008).

Levels of SCN VIP mRNA show a circadian rhythm (Shinohara et al., 1993), which is sexually dimorphic, possibly E2-sensitive, and approximately in antiphase between males and females, with males having higher mRNA levels during nighttime in both nocturnal and diurnal rat species (Krajnak et al., 1998a; Mahoney et al., 2009). GnRH neurons are contacted by VIP-containing fibers in both sexes but the number of contacts may be sexually dimorphic and age-dependent (Horvath et al., 1998; Kriegsfeld et al., 2002). In adult female rodents, about 30–40% of GnRH neurons are contacted by VIP fibers (Beek et al., 1993; Horvath et al., 1998), and ∼40% express the VIP receptor, VPAC2 (aka VIP2), which did not change between morning and afternoon of proestrus (Smith et al., 2000). However, a recent study showed that specific suppression of VPAC2 expression in GnRH neurons alters female mice estrous cycles (Kahan et al., 2023). Functionally, GnRH neurons in females contacted by VIP preferentially expressed c-fos at the time of the GnRH/LH surge (Beek et al., 1994) and infusion of VIP antisense in SCN impairs c-fos expression in these GnRH neurons (Gerhold et al., 2005; Kahan et al., 2023). In addition, a recent study showed that the loss of the VAX1 transcription factor required for normal VIP expression, delays puberty onset, alters females estrous cycle length and potentiates the GnRH response to kisspeptin stimulation (Van Loh et al., 2023). Together, the experimental evidence highlights the importance of VIP signaling, from cells in the SCN to GnRH neurons, for maintenance of normal reproductive function. The aim of the present study was to determine the signaling pathway by which VIP activates GnRH neurons.

## Materials and methods

### Organotypic cultures of GnRH cells in explants

Organotypic cultures, shown to maintain GnRH cells with characteristics similar to GnRH cells recorded *in situ* (Constantin, 2011), were made as previously described (Constantin and Wray, 2008a). Briefly, embryos were obtained from time-mated NIH Swiss females, euthanized by CO_2_ according to the NIH-NINDS ACUC animal ethics guidelines. Nasal pits were then dissected in Gey’s balanced salt solution, supplemented with 5 mg/mL glucose, and adhered onto a Permanox coverslip (Nunc™) using a plasma/thrombin clot. Explants were cultured in a defined serum-free medium (SFM) at 37°C in a humidified incubator with 5% CO_2_. Explants were treated between day 3 and day 6 with SFM containing 2.3 μM fluorodeoxyuridine to inhibit proliferation of dividing neurons and non-neuronal tissue. Thereafter, SFM was replaced every 2–3 days. Explants were used between 7 and 11 days *in vitro* (d *in vitro*) when GnRH secretion can be neuromodulated (Constantin and Wray, 2016). For both calcium imaging experiments and electrophysiological experiments on bursts frequency, pharmacological challenges were performed in the presence of an amino acid blocker cocktail (AAB: bicuculline 20 μM, d-AP5 10 μM, CNQX 10 μM, see section “Drugs” for details) to isolate GnRH neurons from their GABAergic and glutamatergic inputs.

### Calcium imaging

Large number of GnRH cells can be assayed in explants as compared to *in situ* (Jasoni et al., 2010). Since both GnRH neurons in explants and *in situ* exhibit calcium oscillations concomitant with bursts of action potentials (Constantin and Wray, 2008a; Lee et al., 2010), calcium imaging was used to assess GnRH neuronal activity. Calcium imaging was performed using previously established protocols (Constantin and Wray, 2008a,^2016^). Briefly, calcium green-1 AM (Molecular Probes, Eugene, OR) was diluted to 2.7 mM with 20% pluronic F-127/dimethylsulfoxide solution (Molecular Probes) then further diluted down to 13.5 μM with SFM (loading solution), aliquoted, frozen and warmed up just prior use. Explants, maintained at 37°C in humidified atmosphere with 5% CO_2_, were incubated in the loading solution for 20 min then washed in SFM (20 min). After washing, explants were mounted into a perfusion chamber and continuously perfused at ∼300 μL/min with SFM at room temperature (∼25°C) using a low-rate peristaltic pump (Instech Labs, Plymouth Meeting, PA, USA) coupled to a perfusion system (Warner Instruments, Hamden, CT, USA) allowing “in-line” drug treatments. The viability of the cells was assessed at the end with a 40 mM KCl challenge.

**FIGURE 1:**
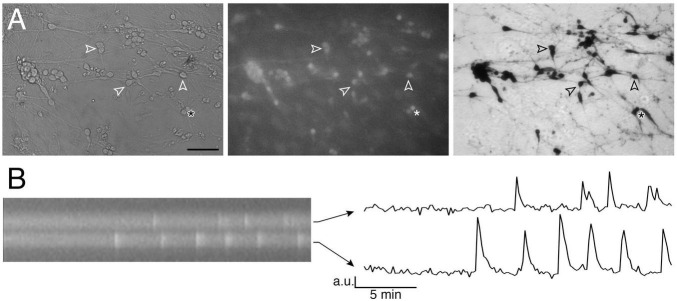
Imaging of GnRH neuronal activity. **(A)** GnRH neurons in organotypic explants were identified by their bipolar cell bodies (left panel), loaded with calcium green-1 AM (middle panel) and their phenotype confirmed with anti-GnRH immunochemistry *post hoc* (right panel; bar 100 μm). Arrowheads point at the same cells throughout the procedure. **(B)** Kymograph (left) from the two cells directly above the asterisk in A (* is showing the location of these 2 cells) and corresponding fluctuating optical densities (arbitrary units, a.u.) i.e., intracellular calcium oscillations (right).

Based on bipolar morphology and location, GnRH-like cells were visualized using an inverted microscope (Eclipse TE2000-E, Nikon), through a 20x fluorescence objective [Fluor 20x; numerical aperture (NA), 0.75; working distance (WD), 1.0 mm] and a charge-coupled device camera (Retiga QImaging, Surrey, Canada) connected to a computer. Time-lapse recording was piloted by iVision imaging software (Scanalytics Inc., Fairfax, VA, USA), and pictures were acquired every 2 s. Excitation wavelengths were provided by a Lumencor LED light engine (Beaverton, OR) by a medium-width excitation bandpass filter at 465–495 nm, and emission was monitored through a 40 nm bandpass centered on 535 nm. The phenotype of the cells recorded was confirmed *post hoc* by immunocytochemistry for GnRH (Figure 1A).

### Electrophysiology

Gonadotropin releasing hormone (GnRH) neurons were recorded in acute brain slices using loose patch clamp. GnRH-GFP male mice (MGI:6158458) (Spergel et al., 1999) were chosen to avoid hormonal fluctuations. Mice were killed by cervical dislocation for burst analysis experiments or anesthetized with isoflurane (VETone, Boise, ID) then decapitated for firing experiments. After cervical dislocation around Zeitgeber time 4.5, the brain was extracted then glued to a vibratome plate and submerged into ice-cold low [Ca^2+^] / high [Mg^2+^] (0.5 mM / 6 mM, respectively) artificial cerebrospinal fluid (aCSF), bubbled with 95% O_2_ / 5% CO_2_. After vibratome sectioning (Leica VT1000S), coronal 200 μm-slices were incubated at 30°C in normal aCSF containing: 118 mM NaCl, 3 mM KCl, 2.5 mM CaCl2, 1.2 mM MgCl2, 10 mM HEPES, 25 mM NaHCO3, and 11–14 mM D-glucose (pH 7.3), bubbled with 95% O_2_ / 5% CO_2_.

Slices were transferred into a recording chamber mounted on an upright microscope (Nikon Eclipse FN1, Tokyo, Japan) and continuously perfused with oxygenated normal aCSF maintained at 28–30°C, at a rate of ∼2 ml/min. GnRH neurons were identified in slices under fluorescence (20 nm narrow bandpass EGFP filter centered at 480 nm) using a 40X water immersion objective (Nikon 40X/0.80 W, WD 2.0) and patched under differential interference contrast through a charge-coupled QImaging Retiga EXi Blue camera (Surrey, Canada) or an ORCA-Fusion Digital CMOS camera (Hamamatsu, Japan), piloted by the open-source software Micro-Manager version 1.4. The pipettes (3–7 MOhm) were backfilled with aCSF. Light negative pressure was applied to obtain low resistance seal (15–40 MOhm) allowing detection of spontaneous spikes. Electrophysiological recordings in voltage-clamp mode were acquired at 10 kHz with a Multiclamp 700B amplifier (Molecular Devices, San Jose, CA) using a lowpass filter and digitized at 1 kHz by a 1550 Digidata analog-to-digital converter (Molecular Devices). A 5-min minimum baseline period was recorded for each cell and cells with unstable activity or silent during this baseline period were not included in the study.

### Drugs

Drugs were kept in 500X-1,000X stock solutions and diluted in SFM prior the experiments. (-)-Bicuculline methochloride (BIC; GABA_*A*_ receptor antagonist), d(-)-2-amino-5-phosphonopentanoic acid (d-AP5; NMDA receptor antagonist), 6-Cyano-7-nitroquinoxaline-2,3-dione (CNQX; AMPA/kainate receptor antagonist) and bisindolylmaleimide X (BIM-X; protein kinase C blocker) were purchased from Tocris (Bristol, UK) or Cayman Chemical (Ann Arbor, MI). SQ 25236 (adenylyl cyclase blocker) and UCL2077 (slow afterhyperpolarization blocker) were obtained from Tocris. TRAM-34 (KCa3.1 blocker) was obtained by MedChemExpress (Monmouth Juntion, NJ, USA). Rp-cAMPS triethylammonium salt (protein kinase A blocker), charybdotoxin (ChTx; fast afterhyperpolarization blocker) and apamin (medium afterhyperpolarization blocker) were obtained from Sigma (Saint-Louis, MO, USA). PG97-269 and PG99-465 (VPAC1 and VPAC2 blockers, respectively) were obtained from Bachem (Torrance, CA, USA). Cholera toxin (CTX) and wortmannin were obtained from Cayman Chemical. Vasoactive intestinal peptide (VIP) was obtained from Phoenix Pharmaceuticals (Burlingame, CA, USA).

### Analysis

Calcium imaging recordings were divided into 3–4 periods, according to treatments. For each GnRH neuron, calcium oscillations were detected over time (Figure 1B) and the frequency of calcium oscillations calculated for each treatment period (peaks/min) as previously described (Constantin and Wray, 2008a). Eighteen paradigms were designed for this project. In average, each paradigm was repeated during ∼5 independent experiments. A total of 2563 cells from 105 explants were analyzed (24.0 ± 1.4 cells/explant).

To avoid the influence of each explant sample size, all the values from the cells (*n*) from one explant were averaged for each period and each explant (*N*) was used as an individual animal value and used for statistical analysis. Statistical analysis was performed using one-way ANOVA. Statistical significance was set at a *p*-value of 0.05. For pretreatments that led to a plateau-type VIP response (BIM-X, ChTx and apamin), the frequency of calcium oscillations was no longer a valid measurement since calcium levels remained high over extended times. As such, the area under the curve (AUC) was measured for two 2-min periods for each cell: one immediately before VIP exposure, another immediately after VIP exposure to capture the plateau. The AUC during VIP was expressed as % of the AUC prior to VIP. Statistical analysis was performed between treatments with a Kruskal–Wallis test, followed by Dunn’s multiple comparisons test. Statistical significance was set at a *p*-value of 0.05.

Electrophysiological recordings were analyzed with Clampfit 10. For the analysis of bursts and intraburst frequency, continuous recordings were divided into 4 periods and action potentials (APs) were detected for each period. The firing pattern was examined to find bursts of APs according to the method previously described (Constantin et al., 2012). Briefly, a burst was defined as a cluster of ≥ 2 APs within 1 s, and must be separated by a period of silence for at least 4 s from the previous AP. The numbers of bursts between consecutive periods were compared using repeated measurement one-way ANOVA. For analysis of mean firing rate (Hertz), events were divided in 10-s bins. A one-way ANOVA was used to compare the average firing rate of a 2-min period before, during and at least 5 min after drug application and a paired *t*-test was used to compare, the two main periods of interest. Significance was determined by *p* < 0.05, and data are presented as mean ± SEM, except for beeswarm plots where SD was chosen to better represent the datapoint spread (through the manuscript *N* & *n* represent the number of animals and cells used, respectively).

### Immunocytochemistry for GnRH

To confirm that recorded cells were GnRH neurons, immunocytochemistry was performed. After each calcium imaging experiment, explants were fixed at room temperature (RT, 1 h) in 0.1 M phosphate-buffered saline, pH 7.4 (PBS), containing 4% formaldehyde. After a wash in PBS, explants were kept in cryoprotectant until immunocytochemistry. On the first day, after thorough washes in PBS, explants were blocked in 10% normal goat serum/0.3% Triton X-100 (RT, 1 h), washed again in PBS then incubated in anti-GnRH rabbit antibody, SW-1 (Wray et al., 1989) (overnight, 4°C). The next day, after washes in PBS, explants were incubated in a biotinylated donkey anti-rabbit antibody (1:500 in PBS/0.3% Triton X-100; Vector Laboratories, Inc., Burlingame, CA) (RT, 1 h) and processed for avidin–biotin horseradish peroxidase/3,3′-diaminobenzidine detection (Figure 1A).

## Results

### VIP increases GnRH neuronal activity via VPAC2 receptor

**FIGURE 2:**
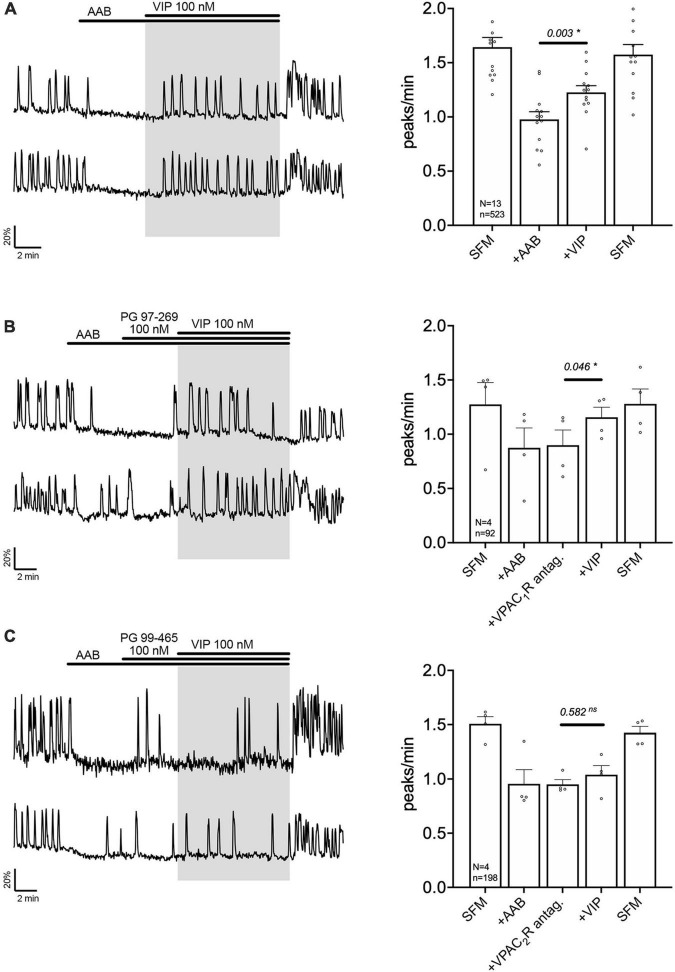
Vasoactive intestinal peptide (VIP) excites GnRH neurons via VPAC_2_ receptor. Left, two representative traces from cells in each condition outlined below. Right, Quantification of GnRH neuronal activity from > 3 independent experiments for each paradigm. **(A)** VIP (100 nM) increased calcium oscillations in cultured GnRH neurons in presence of an amino acid blocker cocktail (AAB). **(B)** VPAC1R blocker (PG 97-269) did not prevent the excitation, whereas VPAC2R blocker did [PG 99-465, **(C)**]. The change in GnRH neuronal activity between pre-VIP period vs. VIP period was assessed using a paired *t*-test (significance, *p* < 0.05, mean ± SEM). Significance (*), *p* < 0.05, mean ± SEM.

Exogenous VIP (100 nM) increased the frequency of calcium oscillations in a subpopulation of GnRH neuron population (Figure 2A). These data are consistent with GnRH neurons electrophysiologically recorded in acute brain slices (Christian and Moenter, 2008; Piet et al., 2016) and imaged in GnRH-GCaMP3 acute brain slices (Piet et al., 2016). To assess which VIP receptor(s) mediated the increase in GnRH neuronal activity, two subtype-specific VIP receptor antagonists were used, PG 97-269 (100 nM) or PG 99-465 (100 nM) for VPAC1 receptor and VPAC2 receptor, respectively (Figures 2B, C). Consistent with the expression data from GnRH cells in adults (Smith et al., 2000), only co-application of the VPAC2 receptor antagonist (PG 99-465, 100 nM) with VIP prevented the increase in the frequency of calcium oscillations (Figures 2B, C). These data indicate a subpopulation of GnRH neurons are excited by VIP via the VPAC2 receptor in our model.

### Both PKA activation and PLC activation/PIP_2_ depletion increase GnRH neuronal activity

**FIGURE 3:**
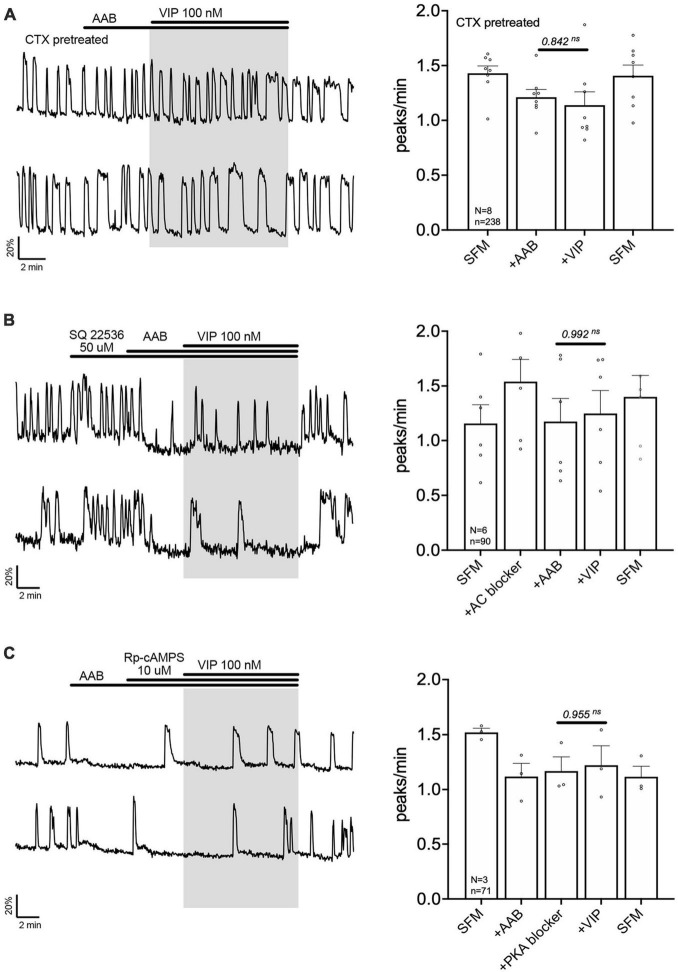
Vasoactive intestinal peptide (VIP)-triggered excitation—signaling via Gs G-protein, adenylyl cyclase and protein kinase A. Left, two representative traces from cells in each condition outlined below. Right, Quantification of GnRH neuronal activity from > 3 independent experiments for each paradigm. Pretreatment with Gs G-protein uncoupler [CTX, cholera toxin 10 ng/mL, > 2 h, **(A)**], adenylyl cyclase blocker [SQ 22536, **(B)**] or protein kinase A blocker [Rp-cAMPS, **(C)**] prevented the VIP (100 nM) response in GnRH neurons. The change in GnRH neuronal activity between pre-VIP period vs. VIP period was assessed using a paired *t*-test (significance, *p* < 0.05, mean ± SEM).

**FIGURE 4:**
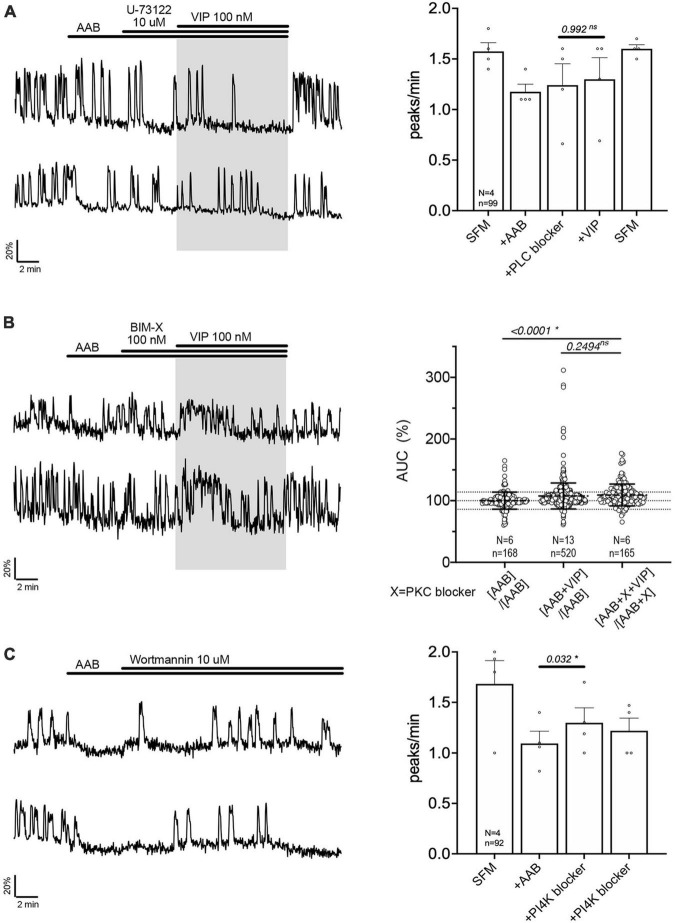
Vasoactive intestinal peptide (VIP)-triggered excitation—signaling relies upon PLC/PIP_2_ depletion, but not PKC activation. Left, two representative traces from cells in each condition outlined below. Right, Quantification of GnRH neuronal activity from > 3 independent experiments for each paradigm, using peak analysis **(A, C)** or AUC analysis **(B)**. Pretreatment with phospholipase C blocker [U-73122, **(A)**], but not protein kinase C blocker [BIM-X, **(B)**] prevented the VIP (100 nM) response in cultured GnRH neurons. Depletion of membrane PIP2 via phosphatidylinositol 4-kinase blocker evoked excitation in GnRH neurons [Wortmannin, **(C)**]. The change in GnRH neuronal activity between pre-VIP period vs. VIP period **(A)** or AAB period vs. wortmannin period **(C)** was assessed using a paired *t*-test (significance, *p* < 0.05, mean ± SEM). The AUC evoked by VIP for all the cells was compared to the AUC with AAB only using Kruskal–Wallis test, followed by Dunn’s multiple comparisons test. Statistical significance was set at a *p*-value of 0.05 (mean ± SD to better visualize the beeswarm spread, dotted lines aligned to mean ± SD for AAB). Significance (*), *p* < 0.05, mean ± SEM.

The signaling pathway by which VPAC2 receptor increases GnRH neuronal activity is unknown. A series of experiment were performed to define this pathway. First, cholera toxin (CTX, 10 ng/mL, for > 2 h prior to exposure to VIP treatment) was used to dissociate the alpha subunit from the beta gamma-subunit complex in the stimulatory G protein (Gs). After CTX treatment, VIP failed to increase GnRH neuronal activity (Figure 3A), indicating VPAC2 mainly couples to Gs in GnRH neurons. To examine whether Gαs stimulated the production of 3′,5′-cyclic adenosine monophosphate (cAMP) which subsequently activated protein kinase A (PKA) (Hao et al., 2006), two blockers were applied prior to VIP, SQ 25236 (50 μM) or Rp-cAMPS (10 μM), adenylyl cyclase blocker and PKA blocker, respectively. In both cases, the pretreatment prevented the VIP-induced increase in the frequency of calcium oscillations (Figures 3B, C), highlighting that the VPAC2 canonical Gαs/cAMP/PKA signaling pathway mediated the increase in GnRH neuronal activity. However, VPAC2 can also activate phospholipase C (PLC) in a pertussis toxin –sensitive and –insensitive manner, i.e., Gi/o and Gq/11, respectively (MacKenzie et al., 1996, 2001). Thus, a PLC blocker (U-73122; 10 μM) was applied prior to VIP. Unexpectedly, this pretreatment also impaired the response to VIP (Figure 4A), indicating that VPAC2 can use both its canonical signaling pathway and a PLC-dependent pathway to increase GnRH neuronal activity.

**FIGURE 5:**
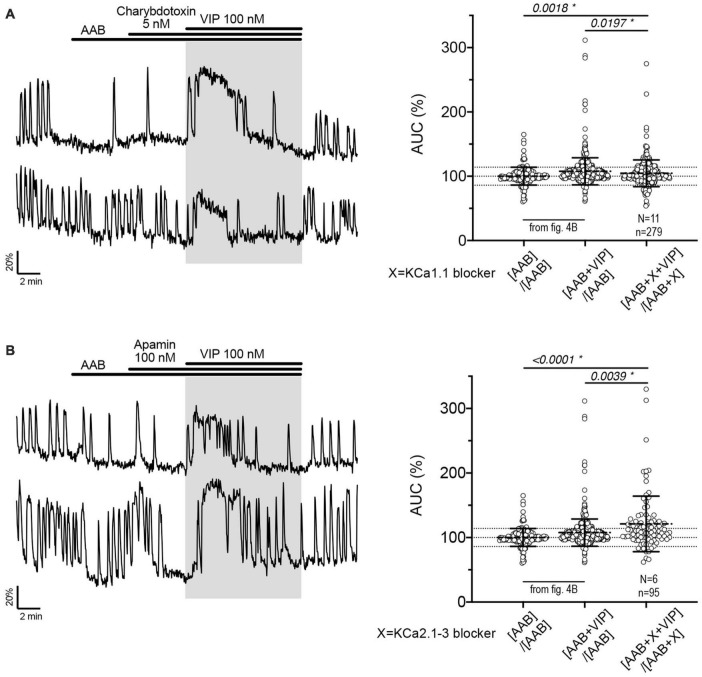
The fast and medium I*_*AHP*_* shape calcium oscillations during the VIP-increased GnRH neuronal activity but do not contribute to the response to VIP itself. Left, Two representative traces showing the plateau-like calcium response to VIP (100 nM) in cultured GnRH neurons pretreated with KCa1.1 blocker [charybdotoxin, **(A)**] or KCa2.1-3 blocker [apamin, **(B)**]. Right, Quantification of GnRH neuronal activity from > 3 independent experiments for each paradigm, using AUC analysis. The AUC evoked by VIP for all the cells was compared to the AUC with AAB only using Kruskal–Wallis test, followed by Dunn’s multiple comparisons test. Statistical significance was set at a *p*-value of 0.05 (mean ± SD to better visualize the beeswarm spread, dotted lines aligned to mean ± SD for AAB). Significance (*), *p* < 0.05, mean ± SEM.

The activation of PLC leads to the formation of second messengers, diacylglycerol (DAG) and inositol 1,4,5-trisphosphate. Since DAG activates the protein kinase C (PKC), a PKC blocker (BIM-X; 100 nM) was applied prior to VIP. The response to VIP was not prevented (Figure 4B, traces). However, cells now responded with an increase in calcium levels detected as a plateau rather than an increase in the frequency of calcium oscillations. Because our peak analysis is based on the quantification of calcium oscillations over time and fails to detect the increase in calcium levels during a plateau, analysis of the area under the curve (AUC) was measured for two 2-min consecutive periods for each cell: one immediately before VIP exposure, another immediately after VIP exposure. Cells remaining in AAB were used to define the spontaneous AUC fluctuation between 2 periods (Figure 4B, beeswarm graph; left dataset in this figure and Figure 5). Cells switched from AAB to AAB+VIP established the classical AUC fluctuation with VIP (middle dataset in this figure and Figure 5). Cells switched from AAB+BIM-X to AAB+BIM-X+VIP AUC analysis detected an increase in AUC with AAB+BIM-X+VIP, compared to AAB alone, and this increase was comparable to AAB+VIP. Thus, the VIP response occurs downstream of PLC activation, through a PKC-independent mechanism. Degradation of membrane PIP_2_ occurs during the formation of DAG and inositol 1,4,5-trisphosphate (Putney and Tomita, 2012) and PIP_2_ modulates channels (Suh and Hille, 2008). To test whether the depletion of the membrane PIP_2_ would be sufficient to excite GnRH neurons, wortmannin (10 μM) was used to block phosphatidylinositol 4-kinase. The decrease in PIP2 synthesis, i.e., depletion of the membrane PIP2, increased GnRH neuronal activity (Figure 4C).

### VIP modulates GnRH neuron excitability by inhibiting UCL2077-sensitive channels, known to be involved in slow I_*AHP*_

Since calcium oscillations reflect bursts of action potentials (Constantin and Wray, 2008a) while calcium plateaus are associated with tonic firing, an increase in the frequency of calcium oscillations should elicit a change in burst patterning. Charybdotoxin-sensitive calcium-activated currents have been suggested to regulate firing (Hiraizumi et al., 2008), and apamin- and UCL2077-sensitive calcium-activated afterhyperpolarization currents (I*_*AHP*_*) are linked to intraburst and interburst patterning, respectively (Lee et al., 2010). Thus, we examined whether calcium-activated hyperpolarizing currents expressed in GnRH neurons (Kato et al., 2006; Hiraizumi et al., 2008; Lee et al., 2010; Zhang et al., 2013) might be the specific target of PKA/PIP_2_ depletion in the VIP-triggered signaling.

**FIGURE 6:**
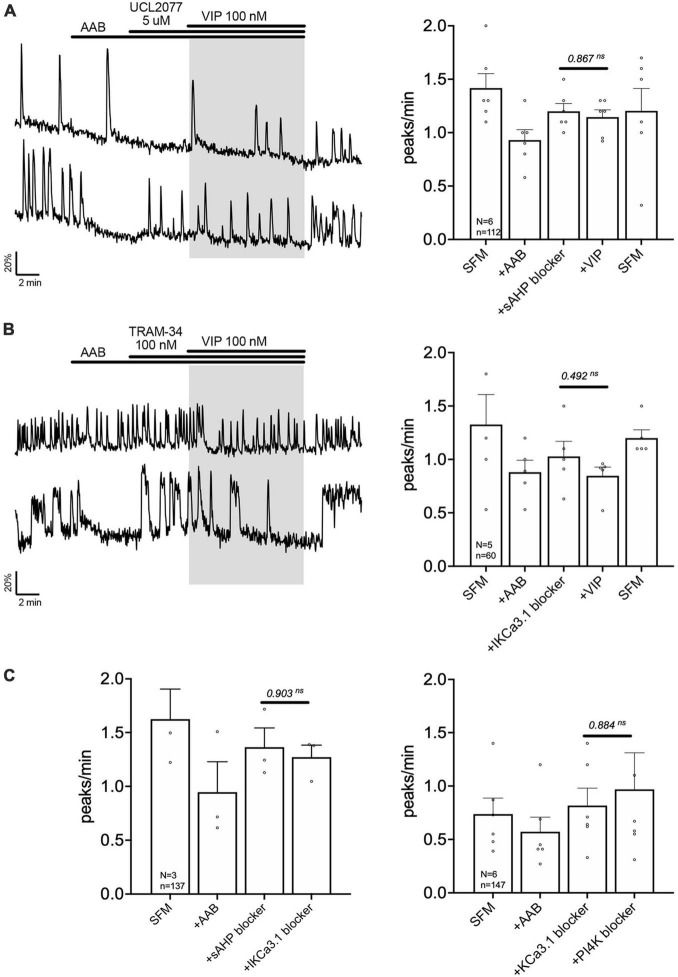
Vasoactive intestinal peptide (VIP) modulates the slow I*_*AHP*_* via inhibition of UCL2077-sensitive KCa3.1 channel. Left, two representative traces showing absence of a calcium response to VIP (100 nM) in GnRH neurons pretreated with slow I*_*AHP*_* blocker [UCL2077, **(A)**] or KCa3.1 blocker [TRAM-34, **(B)**]. Right, Quantification of GnRH neuronal activity from > 3 independent experiments for each paradigm. **(C)** Quantification of GnRH neuronal activity showing that UCL2077 prevented the stimulatory effect of TRAM-34 (left), and that TRAM-34 prevented PIP2 depletion-induced excitation (right). The change in GnRH neuronal activity between pre-VIP period vs. VIP **(A,B)** period or UCL2077 vs. TRAM-34 and TRAM-34 vs. wortmannin period **(C)** was assessed using a paired *t*-test (significance, *p* < 0.05, mean ± SEM).

Charybdotoxin [KCa1.1 channel (fast I*_*AHP*_*) blocker] did not prevent the calcium response to VIP (Figure 5A, traces). As with BIM-X [PKC blocker (see Figure 4B)], a calcium plateau led to a lack of response as defined by our peak analysis. A similar response was seen after application of apamin [KCa2.1-3 channel (medium I*_*AHP*_*) blocker; Figure 5B]. When using the AUC analysis, we detected an increase in AUC with VIP in presence of AAB+charybdotoxin and AAB+apamin, compared to AAB alone. Yet, both responses differed to the AUC response with VIP in presence of AAB (Figures 5A, B, beeswarm graphs). These data indicated that both KCa1.1 and KCa2.1-3 channels play an active role in patterning intraburst spikes (i.e., ending action potential bursts), and subsequently shaping calcium oscillations, during the VIP-triggered signaling but are not the targets downstream of VPAC2. In contrast, UCL2077 (slow I*_*AHP*_* blocker) alone increased the frequency of calcium oscillations, mimicking the VIP-induced excitation (Table 1a, row) and no subsequent response to VIP was observed (Figure 6A). This suggest that VIP increases the frequency of calcium oscillations by inhibition of the UCL2077-sensitive I*_*AHP*_*. The AUC analysis was applied to this dataset and no increase in AUC with AAB+UCL2077+VIP was detected compared to AAB alone. Although bicuculline has been previously shown to block SK channels (Khawaled et al., 1999), in the present experiments selective channels inhibitors for slow I*_*AHP*_* were always applied after recording of the baseline calcium activity in bicuculline, and the subsequent effect compared to this baseline. In our hand, all slow I*_*AHP*_* inhibitors used had an additional effect, indicating a higher specificity for their targets than bicuculline.

**TABLE 1:** Frequencies of calcium oscillations in GnRH neurons.

	Drug during periods 3 and 4	Period 1 SFM	Period 2 AAB	Period 3 —	Period 4 —	Period 5 SFM	*p*-value (between periods 3 and 4)	Animals, cells (*N*, *n*)
		Frequencies in peaks/min						
a	sAHP blocker: UCL2077	1.20 ± 0.23	0.83 ± 0.22	1.10 ± 0.21	1.10 ± 0.21	1.10 ± 0.27	0.0275*	5, 77
b	Kv7 blocker: XE991	1.20 ± 0.16	0.95 ± 0.27	0.82 ± 0.19	0.71 ± 0.20	0.89 ± 0.19	0.4265^ns^	4, 76
c	Kir6 blocker: glibenclamide	1.40 ± 0.12	0.89 ± 0.09	0.89 ± 0.14	0.70 ± 0.14	1.10 ± 0.12	0.9859^ns^	5, 30
d	KCa3.1 blocker: TRAM-34	1.50 ± 0.17	0.98 ± 0.22	1.10 ± 0.21	1.20 ± 0.17	1.30 ± 0.12	0.0036*	6, 154

Summary of the control experiments testing the effects of drugs alone over time. Only the 2 consecutive periods (period 3 and period 4) were analyzed for changes in the frequency of calcium oscillations (paired *t*-test, significance, *p* < 0.05). The dose for each drug is as follow: AAB [BIC 20 μM, CNQX 10 μM, d-AP5 10 μM], UCL [5 μM], XE991 [10 μM], glibenclamide [10 μM], TRAM-34 [100 nM]. Significance (*), *p* < 0.05, mean ± SEM.

Literature support these data since PKA-dependent phosphorylation increases the frequency of calcium oscillations (Constantin and Wray, 2008b) and inhibits slow afterhyperpolarization currents in GnRH neurons (Zhang et al., 2013), and PIP_2_ membrane content modulates a variety of ion channels (Suh and Hille, 2008), including those involved in the slow I*_*AHP*_* (Villalobos et al., 2011; Kirchner et al., 2017).

### VIP modulates GnRH neuron excitability by inhibiting KCa3.1 and possibly slow I_*AHP*_

The channels contributing to the slow I*_*AHP*_* are likely multiple and cell-specific (Hsu et al., 2020). Among the putative players are the potassium channels KCa3.1, Kir6 and Kv7. Since the blockers of Kir6 and Kv7, XE991 (10 nM) and glibenclamide (10 μM), respectively, had no effect upon the frequency of calcium oscillations in GnRH neurons (Table 1b, c), the contribution of KCa3.1 channels to the slow I*_*AHP*_* was tested with a specific blocker, TRAM-34 (100 nM). Like UCL2077, TRAM-34 alone increased the frequency of calcium oscillations (Table 1d) and no subsequent effect of TRAM-34 was observed when applied after UCL2077 (Figure 6C left), supporting the fact that both drugs share the same target. In addition, like UCL2077, TRAM-34 prevented the subsequent response to VIP (Figure 6B) and AUC analysis revealed no increase in the AUC with AAB+TRAM34+VIP compared to AAB alone. Together, these data indicate a contribution of KCa3.1 channel in the slow I*_*AHP*_* in GnRH neurons and that VIP increases the frequency of calcium oscillations by inhibition of this channel. Literature supports that KCa3.1 can be suppressed by PKA phosphorylation (Wong and Schlichter, 2014; Tiwari et al., 2019) and by membrane phospholipid depletion (Srivastava et al., 2005). As such, the effect of the KCa3.1 blocker, TRAM-34 (100 nM), on the wortmannin (phosphatidylinositol 4-kinase blocker)-induced stimulation (see Figure 4C) was tested. This pretreatment prevented the increase in GnRH neuronal activity (Figure 6C right) consistent with TRAM-34 blockade.

### KCa3.1 blockers prevent the response of GnRH neurons to VIP in adult brain slices, consistent with its contribution to slow I_*AHP*_

**FIGURE 7:**
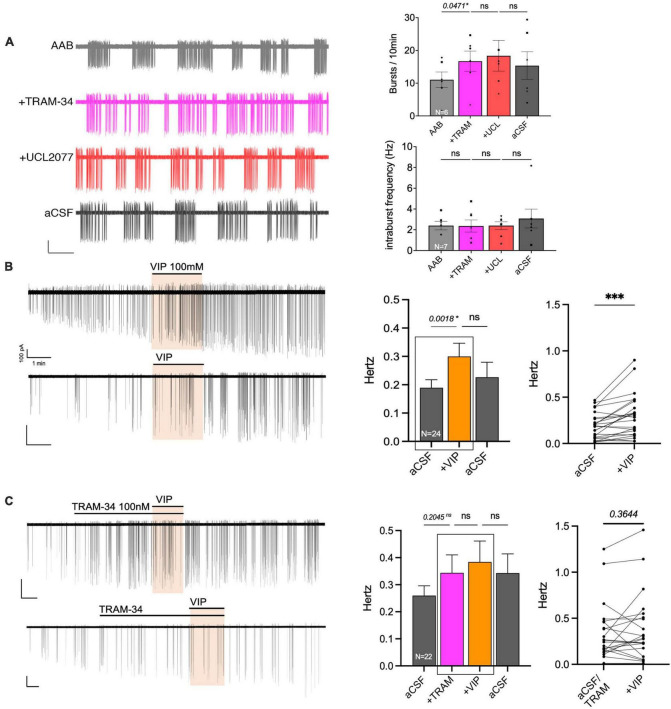
The KCa3.1 blocker, TRAM-34, mimics the effects of UCL2077 on GnRH neuron burst occurrence in acute slices and inhibits VIP response. **(A)** Left, representative trace showing effect of TRAM-34 then UCL2077 on AAB-treated GnRH neuron burst occurrence (bars: 20 pA, 30 s). Right, Quantification of two burst parameters during subsequent drug addition, burst occurrence (bursts/10 min; top) & intraburst frequency (bottom; 6 cells from 6 animals). The change in burst parameters between consecutive periods was assessed using a repeated measurement one-way ANOVA (significance, *p* < 0.05). **(B,C)** Left, two representative traces showing the increase in spontaneous firing induced by VIP [100 nM, **(B)**] in GnRH neurons from mouse brain slices and traces showing the absence of VIP-induced excitation with addition of KCa3.1 blocker prior to VIP [TRAM-34 100 nM, **(C)**]. Middle, histogram showing the quantification of mean firing rate from 24 [VIP, **(B)**] or 22 [TRAM-34, VIP, **(C)**] GnRH cells (mean ± SEM, statistical significance was assessed using a one-way ANOVA). Right, before-after plot showing individual data corresponding to the 2 recording periods squared in middle histogram. Statistical significance was assessed using a paired *t*-test, *p* < 0.0001*** to confirm increased firing rate with VIP but not in presence of TRAM-34. Significance (*), *p* < 0.05, mean ± SEM.

Data from GnRH neurons in explants indicated that VIP targeted the UCL2077-sensitive target, known to be involved in slow I*_*AHP*_*, and specifically suggested the contribution of KCa3.1. In acute brain slices, the effect of UCL2077 on burst patterning in GnRH neurons is linked to its action upon the slow I*_*AHP*_* (Lee et al., 2010), increasing burst occurrence but not intraburst frequency. Yet, the identity of the channel involved in the GnRH neuron slow I*_*AHP*_* is unknown. Thus, the contribution of KCa3.1 upon burst patterning was examined in GnRH neurons in acute slices using TRAM-34 (KCa3.1 blocker). In presence of AAB, TRAM-34 (200 nM) was applied followed by UCL2077 (10 μM, slow I*_*AHP*_* blocker) to assess the effects on burst occurrence and intraburst frequency in spontaneously firing GnRH neurons (Figure 7A). The hypothesis was as follow: If TRAM-34 and UCL2077 shared the same target, TRAM-34 should increase the burst occurrence and the addition of UCL2077 should have no additional effect. In contrast, if TRAM-34 and UCL2077 do not share the same target, TRAM-34 might increase the burst occurrence and /or intraburst frequency but the addition of UCL2077 should have an additional effect on burst occurrence but not intraburst frequency. TRAM-34 increased burst occurrence and adding UCL2077 did not increase it further (Figure 7A top). Neither TRAM-34 nor UCL2077 modified the intraburst frequency (Figure 7A bottom). These data are consistent with the first scenario, suggesting TRAM-34 and UCL2077 share the same target. We then confirmed that the inhibition of KCa3.1 prevents the VIP-induced stimulation of GnRH neurons in slices from adult brains. Similar with the results from the explant model (Figures 2A, 6B), VIP (100 nM) significantly increased the firing activity of GnRH neurons in brain slices (Figure 7B). Although TRAM-34 alone (100 nM) triggered variable effects upon GnRH neurons firing rate, many cells showed an increase in spontaneous firing and TRAM-34 prevented any further effect of VIP (Figure 7C). Together, these data on GnRH neurons in acute brain slices indicate that VIP excites GnRH neurons by inhibiting a component sensitive to UCL2077 and TRAM-34. Since UCL2077 is known to impact the slow IAHP in GnRH neurons (Lee et al., 2010), KCa3.1 becomes a potential player in this current.

## Discussion

**FIGURE 8:**
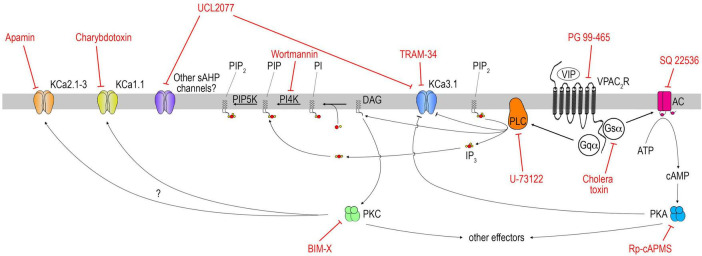
Schematic of VIP signaling pathway in GnRH neurons. Note this schematic aims at summarizing the conclusions reached in this study and does not provide an exhaustive pharmacological profile of each drug. Thus, only the main target(s) of each drug are represented; others deemed out of the scope of this study are not. For instance, charybdotoxin and TRAM-34 can impact some Kv1.x channels. At higher dose, charybdotoxin can also impact KCa3.1.

Vasoactive intestinal peptide (VIP) is an important component of the SCN which relays circadian information to neuronal populations, including GnRH neurons. VIP signaling is involved in the generation and/or timing of the GnRH/LH surge in females (Harney et al., 1996; Kahan et al., 2023), and reproductive behavior in males (Kukino et al., 2022). However, little is known about the signaling pathway activated downstream of the VIP receptor in GnRH neurons (Christian and Moenter, 2008; Piet et al., 2016). Here we show that VIP excites GnRH neurons via VPAC2 and that this excitation relies upon Gs coupling of the receptor, the production of cAMP by adenylyl cyclase and the activity of PKA, but also Gq/PLC activation (Figure 8). Notably, the slow I*_*AHP*_* blocker (UCL2077) prevented the response to VIP. To our knowledge, this is the first time that a physiological ligand, VIP, targets the UCL2077-sensitive slow I*_*AHP*_* in a protein kinase A- and PLC-dependent manner in GnRH neurons.

The role of circadian signals via VIP has been extensively studied in females but to a much lesser extent in males. In females, when circadian signals and estradiol levels coincide, E2 feedback switches from negative to positive and a GnRH surge occurs, triggering ovulation. Although anteroventral periventricular nucleus (AVPV) Kiss1-expressing neurons are a critical component for the GnRH surge (Clarkson et al., 2008), Kisspeptin administered by intracerebroventricular (ICV) injection or by microdialysis cannot advance the onset of the LH surge in intact or OVX+E2 rats (Roa et al., 2008). Furthermore, GnRH neurons exhibit daily changes in their sensitivity to intraperitoneal kisspeptin stimulation (Williams et al., 2010). Thus, other factor(s) must tune GnRH neuron responsiveness to kisspeptin. E2 regulates GnRH neuron ion channels directly (Bálint et al., 2016) and downstream of the Kisspeptin receptor via ERβ (Bosch et al., 2013), however mice with GnRH neuron-specific deletion of ERβ exhibit normal cycles (Cheong et al., 2014). Together, these data indicate that other signaling pathways within GnRH neurons are essential for the surge to occur.

Both brain muscle Arnt-like 1 (BMAL1) and period-2 (PER2) proteins cycle with a circadian period in GnRH neurons (Hickok and Tischkau, 2010). However, mice with GnRH neuron-specific deletion of *Bmal1* exhibit normal cycles (Tonsfeldt et al., 2019), despite a slight delay of the LH surge (Bittman, 2019). Neurons expressing RFamide-related peptide 3 (RFRP-3), mammalian equivalent to the avian gonadotropin-releasing inhibitory, innervate GnRH neurons (Kriegsfeld et al., 2006). In female Syrian hamsters, RFRP-3 neurons receive inputs from the SCN (Russo et al., 2015). Elegant experiments in “split” hamsters show expression of c-fos in RFRP-3 neuronal and GnRH neuronal subpopulations in opposite hemispheres at the time of the LH surge, indicating disinhibition of a GnRH neuronal subpopulation driving the LH surge (Gibson et al., 2008). The expression of c-fos in RFRP-3 neurons, lower during the afternoon, can be decreased further with ICV VIP, yet RFRP-3 neurons do not express VIP receptors (Russo et al., 2015). In mice, the role of RFRP-3 in the timing of the LH surge is less clear. While RFRP-3 might contribute to the timing of the LH surge (Poling et al., 2017), females lacking the receptor for RFRP-3 exhibit a normal LH surge (Leon et al., 2014) and in rats, ICV VIP does not change c-fos expression in RFRP-3 neurons (Kauffman et al., 2014). Therefore, VIP inputs from the SCN directly onto GnRH neurons, long ago identified (Beek et al., 1993), remain a candidate for gating kisspeptin responsiveness and onset of the LH surge. A recent study showing greater GnRH sensitivity to kisspeptin when VIP expression is abnormal, further supports this hypothesis (Van Loh et al., 2023).

While reproductive senescence impairs the pulse generator in both sexes (Steiner et al., 1984; Wise et al., 1988), it also affects the LH surge and can help dissect its components. In middle-aged OVX+E2 rats, the LH surge is dampened and delayed (Cooper et al., 1980). Microdialysis of kisspeptin restores the amplitude of the LH surge, but not its timing (Neal-Perry et al., 2009). On the other hand, aging also impacts the circadian rhythm of VIP mRNA levels in the SCN (Krajnak et al., 1998b). VIP-contacted GnRH neurons fail to express c-fos in middle-aged rats, suggesting a decreased sensitivity of GnRH neurons to VIP inputs (Krajnak et al., 2001) and ICV VIP restores the amplitude and the timing of the LH surge in middle-aged rats (Sun et al., 2012), in an AVPV Kisspeptin–independent manner (Kauffman et al., 2014). Thus, the VIP rescue of the LH surge in middle-aged animals might, in fact, rely only on GnRH neurons and highlights the importance of understanding this signaling pathway.

To further unravel the VIP-GnRH interaction, this study investigated the signaling pathway activated downstream of the VIP receptor in GnRH neurons. First, we showed, in a model devoid of brain that VIP increases the frequency of calcium oscillations in primary GnRH neurons, via VPAC2 receptor, and that this excitation relies upon Gs coupling of the receptor, the production of cAMP by adenylyl cyclase and PKA signaling, and also PLC activation. Both signaling pathways have been reported in non-GnRH neurons downstream of VPAC2 (Mazzocchi et al., 2002; Langer, 2012) and both operate together downstream of VPAC1 (Hu et al., 2011). Calcium oscillations in GnRH neurons rely upon the patterning of action potentials and I*_*AHP*_* play an important role in this patterning (Constantin and Wray, 2008a; Lee et al., 2010). GnRH neurons exhibit charybdotoxin-, apamin- and UCL2077-sensitive I*_*AHP*_* (Hiraizumi et al., 2008; Lee et al., 2010; Zhang et al., 2013). The apamin- and the UCL-sensitive I*_*AHP*_* regulate the intraburst and interburst patterning, respectively (Lee et al., 2010). Since charybdotoxin-sensitive (BK, KCa1.1) and apamin-sensitive (SK, KCa2.1-3) channels contribute to the fast and medium I*_*AHP*_*, respectively (Larsson, 2013), their inhibition would disable the intraburst break, leading to a dysregulation of the calcium response to VIP. In contrast, the slow I*_*AHP*_* blocker, UCL2077, should disable the interburst break, mimic the VIP stimulation but prevent the subsequent effect of VIP. Our observations are consistent with both predictions. This is in agreement with data from neocortical slices in which VIP induces increased cAMP levels, PKA activity and reduces the amplitude of the slow I*_*AHP*_* (Hu et al., 2011). In addition, the inhibitory effect of PLC activation / PIP_2_ depletion on slow I*_*AHP*_* has been described in oxytocin neurons of the supraoptic nucleus (Kirchner et al., 2017). Finally, in CA1 pyramidal neurons, VIP reduces slow I*_*AHP*_* via both PKA activation- and PLC activation/PIP_2_ depletion- dependent mechanisms, independently of PKC activity (Taylor et al., 2014).

The ion channels contributing to the slow I*_*AHP*_* are likely multiple [KCNQ, K(ATP), KCa3.1, and others] and cell-specific (Larsson, 2013). While UCL2077 blocks slow I*_*AHP*_*, it is not a selective compound (Hsu et al., 2020). In GnRH neurons, the slow I*_*AHP*_* are apamin- and kisspeptin- sensitive, and the kisspeptin-sensitive fraction subdivides into UCL2077- sensitive and insensitive (Lee et al., 2010; Zhang et al., 2013). The slow I*_*AHP*_* is disrupted by kisspeptin-activated protein kinase C and by forskolin-activated protein kinase A (Zhang et al., 2013). Here we show the UCL2077-sensitive target, known to contribute to the slow I*_*AHP*_*, is regulated in a protein kinase A- and PLC- dependent manner, is necessary for VIP signaling in GnRH neurons. Furthermore, our data indicate that the UCL2077 and TRAM-34 share the same target, i.e., KCa3.1 channel, consistent with a role of the slow I*_*AHP*_* in GnRH neurons. KCa3.1 channel is known to play a role in the slow I*_*AHP*_* in hippocampal and neocortical neurons (King et al., 2015; Roshchin et al., 2020), to be suppressed by PKA phosphorylation (Wong and Schlichter, 2014; Tiwari et al., 2019) and by membrane phospholipid depletion (Srivastava et al., 2005).

Overall, the importance of the SCN for ovulation is obvious in rodents (Wiegand and Terasawa, 1982; Swann and Turek, 1985). Yet, VIP- or VPAC2- null mice remain fertile (Harmar et al., 2002; Colwell et al., 2003) despite asynchronous SCNs (Aton et al., 2005). The signal triggering ovulation at the appropriate time of the day is evident in species with short estrous cycle like rodents (Barofsky and Harney, 1983; McElhinny et al., 1999; Piet, 2023). However, studies show that ovulation occurs at the end of the night or early morning in a majority of women, phase-locked with the circadian cortisol peak (Cahill et al., 1998; Kerdelhue et al., 2002). In addition, the importance of the circadian clock appears with the association between shift-work and reproductive dysfunction (Simonneaux et al., 2017; Stock et al., 2019). Notably, the importance of circadian cues extends beyond the estrous cycle. A recent study monitoring LH levels in girls and boys showed an increase in LH occurring only at night in both sexes (Demir et al., 2023), consistent with a circadian component involved in postnatal development of the reproductive system (Turek, 1994). VIP inputs from the SCN directly onto GnRH neurons, remain a candidate for modulating the LH surge in females, and pubertal onset and reproductive behavior in both sexes. Dissecting the VIP response pathway in GnRH neurons showed that the UCL2077-sensitive slow I*_*AHP*_* is important for VIP signals to excite GnRH neurons, key components in the HPG axis.

## Data availability statement

The original contributions presented in this study are included in this article/supplementary material, further inquiries can be directed to the corresponding author.

## Ethics statement

The animal study was approved by the NIH-NINDS ACUC animal ethics guidelines. The study was conducted in accordance with the local legislation and institutional requirements.

## Author contributions

SC: Conceptualization, Data curation, Formal Analysis, Investigation, Methodology, Project administration, Supervision, Validation, Visualization, Writing – original draft, Writing – review & editing. CQ: Data curation, Formal Analysis, Investigation, Validation, Visualization, Writing – original draft, Writing – review & editing. KP: Data curation, Writing – original draft. DS: Data curation, Writing – original draft. SW: Conceptualization, Funding acquisition, Investigation, Methodology, Project administration, Resources, Supervision, Validation, Visualization, Writing – original draft, Writing – review & editing.
